# Impact of adjuvant chemotherapy on the survival of patients with breast cancer diagnosed by screening

**DOI:** 10.1002/cam4.2488

**Published:** 2019-09-24

**Authors:** Irene Zarcos‐Pedrinaci, Maximino Redondo, Javier Louro, Francisco Rivas‐Ruiz, Teresa Téllez, Diego Pérez, Francisco Medina Cano, Kenza Machan, Laia Domingo, Maria Mar Vernet, Maria Padilla‐Ruiz, Xavier Castells, Antonio Rueda, María Sala, REDISSEC‐CaMISS Group

**Affiliations:** ^1^ Research Unit, Costa del Sol Hospital Research Network on Health Services in Chronic Diseases (REDISSEC) University of Málaga Marbella Spain; ^2^ Department of Oncohaematology Costa del Sol Hospital Marbella Spain; ^3^ Department of Epidemiology and Evaluation, Hospital del Mar Research Network on Health Services in Chronic Diseases (REDISSEC) Barcelona Spain; ^4^ Department of Surgery Costa del Sol Hospital Research Network on Health Services in Chronic Diseases (REDISSEC) University of Málaga Marbella Spain; ^5^ Department of Radiology Costa del Sol Hospital Marbella Spain; ^6^ Department of Gynaecology Hospital del Mar Barcelona Spain; ^7^ Department of Medical Oncology Hospital Regional Universitario Carlos Haya Málaga Spain

**Keywords:** adjuvant chemotherapy, breast cancer, breast screening, survival

## Abstract

The aim of this study is to determine the survival of patients with breast cancer treated with adjuvant chemotherapy (ACh) after the diagnosis by screening, taking comorbidity into account. This multicenter cohort study examined a population of patients taking part in four national screening programs for the early detection of breast cancer (localized or locally advanced), during the period 2000‐2008. Of the 1248 cancers detected, 266 were prevalent (21.3%), 633 were incident (50.7%), and 349 were interval (27.9%). No significant differences were detected between the three groups in terms of the distribution of comorbidity according to the CCI. After a median follow‐up of 102 months, 22.1% of the patients with interval cancer had died. The corresponding figures for the incident and prevalent cancers were 10.4% and 7.9%, respectively (*P* < .001). The adjusted Cox regression analysis by the stage, CCI and group revealed no differences in the risk of recurrence between the different groups according to the ACh performed. However, there were significant differences in the overall survival; for the interval cancer group without ACh, the risk of death was higher (Hazard ratio: 2.5 [1.0‐6.2]) than for the other two groups. However, for the prevalent and incident groups that did not receive ACh, there was no greater risk of death. This study shows that adjuvant chemotherapy seems to benefit patients with interval breast cancer, who have a poorer prognosis than those with prevalent or incident cancer. However, the role of ACh is unclear with respect to prevalent and incident cancers when comorbidity is taken into account.

## Graphical abstract

Adjuvant chemotherapy seems to benefit patients with interval breast cancer, who have a poorer prognosis than those with prevalent or incident cancer. However, the role of adjuvant chemotherapy is unclear with respect to prevalent and incident cancers when comorbidity is taken into account.

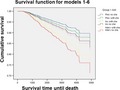

## 1 INTRODUCTION

Breast cancer is the malignant tumor most commonly diagnosed in women in North America and Europe, with approximately 1 151 000 new cases per year (22.7% of all cancer cases among the female population)^1^ and the most prevalent tumor worldwide for 5 years, among both sexes (19.2%). In Spain, the GLOBOCAN 2012 report estimated that the overall incidence of breast cancer was 27 182 cases in 2012. Among women, this tumor presented the highest incidence, mortality, and prevalence for 5 years (29%, 15.5%, and 40.8%, respectively).^2^ Though the mortality from breast cancer increased sharply from 1950s to 1980s,^3^ in most developed countries it has fallen in the last two decades, due to the success of new treatments and the implementation of screening programs^4^.

The breast cancer tumors detected by the screening generally have a good prognosis, due to the existence of biological differences associated with reduced aggressiveness and better survival compared to symptomatic tumors, such as positivity for the expression of hormonal receptors and a lower rate of cellular proliferation.^5^, ^6^

However, this better prognosis is also due to associated biases such as selection bias, lead‐time bias, duration bias, and possibly overdiagnosis bias.^7^, ^8^

Cancer patients often present associated comorbidities, which are known to influence the disease prognosis and to limit the possibilities for the oncological treatment. The Charlson comorbidity index (CCI), a prognostic index that has been validated for the use in various populations, classifies and scores 19 patient‐associated diseases, and has been proposed as an indicator of the probability of 10‐year survival.^9^

The administration of adjuvant chemotherapy (ACh) has been shown to increase the survival in localized breast cancer with clinical risk features such as axillary involvement, large tumor size, the presence of a triple‐negative phenotype or HER2‐enriched subtype, or classification as a tumor with high risk of recurrence according to a genetic platform.^10^, ^11^ However, some tumors present intermediate genetic risk, or have a less aggressive immunohistochemical profile, and these are less likely to benefit from the ACh treatment, which inevitably provokes a deterioration in the patient's quality of life. Some patients who fit this profile are identified in early‐detection programs. In view of the good prognosis associated with the majority of breast cancers diagnosed by screening, we consider it of interest to include this diagnostic factor in the decision‐making process. Accordingly, this study analyses the survival benefit of ACh in each of the patient's groups diagnosed by screening, taking observed comorbidity into account.

## 2 MATERIALS AND METHODS

In breast cancer diagnosed by screening, three groups of patients can be distinguished: Prevalent cancer, diagnosed in the first round, with an apparently better prognosis; incident cancer, diagnosed in subsequent rounds; and interval cancer, detected between one round and the next, by clinical indications.

### 2.1 Study design

This multicenter retrospective cohort study was conducted to identify and classify the breast cancers detected by the mammographic screening as prevalent, incident, or interval tumor.

### 2.2 Study population

This study included 1248 women aged 45‐69 years who had taken part in four national breast‐cancer screening programs, providing biannual mammograms and annual examinations for women with clinical indications of increased risk. They were healthy women and initially without known risk factors for breast cancer. The hospitals involved were all in Spain––in the Costa del Sol (Marbella), the Canary Islands, Sabadell, and Gerona––and the diagnoses and surgical interventions all took place during the period 2000‐2008, with follow‐up to 2014. The women in the study population were mainly derived from the retrospective cohort of the CAMISS study (n = 1086) and the rest had been diagnosed within the screening program provided at the Costa del Sol Hospital, Marbella.^12^

The following inclusion criteria were applied:

- Anatomic‐pathological diagnosis of infiltrating breast cancer.
- Localized or locally advanced stage.
- Age 50‐69 years.

These exclusion criteria were applied:

- The presence of lymphoma, sarcoma, or inflammatory carcinoma.
- Cancer not unresectable.
- Patients who had not received prior chemotherapy, due to associated comorbidity.

### 2.3 Ethical issues

The study was performed in accordance with the good clinical practice guidelines of the Helsinki Declaration. Informed consent was obtained from all patients to take part in the study and for their clinical records to be reviewed. This project was approved by the ethics review board of each of the participating centers.

### 2.4 Variables

The following variables were obtained from the patients’ clinical record.

Main study variable (dependent):

- Variables related to survival (recurrence, metastasis, and death).

Independent variables:

- Treatment: Neoadjuvant: radiotherapy (and date performed); chemotherapy––schedule, number of cycles, and date performed; molecular therapy and date performed; hormonal systemic treatment, and date performed. Adjuvant: reintervention; systemic hormonal treatment and date performed; radiotherapy and chemotherapy, and date performed––schedule, number of cycles, and date performed; molecular therapy and date performed; consultation with rehabilitation; contralateral prophylactic mastectomy; plastic reconstruction; prevention and treatment of treatment complications such as lymphedema or bone loss.
- Patient's clinical history: surgical interventions; patient‐associated diseases, required to calculate the CCI, namely myocardial infarction, congestive heart failure, peripheral vascular disease, cerebrovascular disease, dementia, chronic lung disease, connective tissue pathology, ulcerative disease, mild, moderate or severe hepatic disease, diabetes, diabetes with organic lesion, hemiplegia, renal pathology (moderate or severe), solid neoplasms, leukaemia, malignant lymphoma, solid metastasis, and/or AIDS.^9^

Adjustment variables:

- Patients: age, family history, and menopause.
- Hospital care: the time between diagnosis and first treatment.
- Surgical intervention: date, type of surgical technique (conservative or radical, lymphadenectomy or selective sentinel lymph node biopsy).
- Tumor screening characteristics: those diagnosed in the first round, prevalent; those diagnosed in subsequent rounds, incident; those detected between one round and another, interval; classified by clinical signs, histological type, degree of differentiation, location, size, presence and location of distant metastasis, vascular/nervous infiltration, hormonal receptors, expression of c‐ERB‐b2, number of lymph nodes analyzed, number of positive lymph nodes, involvement of margins, cTNM, pTNM, p53, Ki67, apoptotic index, and selective sentinel lymph node biopsy. The tumors are classified into four immunophenotypes according to the expression of hormone receptors and HER2: Luminal A, Luminal B, HER2+, and TNBC.
- Clinical follow‐up: readmission, recurrence (yes/no, type, and date), treatment complications, status at the end of follow‐up, and death (yes/no, date, and cause).

### 2.5 Statistical analysis

#### 2.5.1 Univariate analysis

Descriptive analysis segmented by the types of breast cancer. Comparison of frequencies between two variables (clinical‐pathological and molecular) by the chi‐square test when appropriate, otherwise (if results <5 expected in >20% of cases) by the Fisher's test. Comparison of the means by the Student's *t* test, after confirming the normal distribution of the quantitative variable and the homogeneity of the variance. If these conditions were not met, the variable was transformed or nonparametric tests were performed.

#### 2.5.2 Multivariate analysis

Cox regression analysis, crude and multivariate, was applied to estimate the risk of recurrence and death. The multivariate analysis was adjusted with entry criteria for the following variables: chemotherapy group (with or without ACh), stage, and comorbidity according to the CCI.

To preserve the statistical power in the multivariate analysis, and since it is an explanatory model, we decided to discard those variables that had a high number of losses (Ki 67 expression), that could be correlated with other variables (comorbidity, estrogen and progesterone receptors, and Her2 expression) or directly did not contribute to the model in the crude analysis (age). The relative risk and the corresponding 95% CI were calculated. In the survival study, the primary endpoint was time elapsed until local recurrence, distance, or death from breast cancer, from the time of diagnosis. Survival times for patients who were still alive or who died from other causes were centered at the date of the last follow‐up. For the Cox regression analysis of the risk of recurrence and death, crude and multivariate analyses were performed (columns 1 and 2, Table 2). The sample sizes for these analyses were somewhat lower, 1028 and 1024, respectively, due to losses to follow‐up.

## 3 RESULTS

Studied data were analyzed for 1248 women aged 45‐69 years, most of whom (n = 1086) formed part of the retrospective cohort of the CAMISS study. Of this population, 45.6% had received adjuvant chemotherapy, 10% neoadjuvant chemotherapy, and 75.7% hormone therapy. Table 1 shows the differences in the distribution of clinical‐biological characteristics between the three groups that received breast cancer screening. In the interval group, 25.7% of the patients presented stage III. The corresponding values for the incident and prevalent groups were 6.8% and 5.8% (*P* < .001), respectively.

**Table 1:** Distribution of frequencies, by screening groups

	Prevalent	Incidence	Interval	
	N = 266 (%)	N = 633 (%)	N = 349 (%)	*P*‐value
Stage				
IN SITU	37 (14.3)	70 (11.3)	14 (4.2)	<0.001
I	139 (53.7)	345 (55.6)	89 (26.6)	
II	68 (26.3)	164 (26.4)	145 (43.4)	
III	15 (5.8)	42 (6.8)	86 (25.7)	
Unknown	7	12	15	
Mean age (min‐max)	55.6 (49‐70)	60.1 (48‐70)	57.7 (49‐71)	<0.001
Phenotype				
Luminal A	60 (48.8)	218 (52.5)	140 (45.5)	<0.001
Luminal B	49 (39.8)	131 (31.6)	84 (27.3)	
HER2	12 (9.8)	28 (6.7)	34 (11)	
Triple negative	2 (1.6)	38 (9.2)	50 (16.2)	
Unknown	143	218	41	
Comorbidity				
Absent	147 (78.2)	387 (70)	254 (73.6)	0.081
Present	41 (21.8)	166 (30)	91 (26.4)	
Unknown	78	80	4	
CCI (min‐max)	0.62 (0‐9)	0.81 (0‐13)	0.79 (0‐7)	0.367
Oestrogen receptors				
Negative	41 (16.1)	100 (16.1)	97 (27.8)	<0.001
Positive	213 (83.9)	520 (83.9)	252 (72.2)	
Unknown	12	13	0	
Progesterone receptors				
Negative	67 (26.4)	199 (32.1)	147 (42.2)	<0.001
Positive	187 (73.6)	420 (67.9)	201 (57.8)	
Unknown	12	14	1	
Her2				
Negative	108 (80.6)	357 (80.6)	245 (77.5)	0.557
Positive	26 (19.4)	86 (19.4)	71 (22.5)	
Unknown	132	190	33	
ki67 expression				
≤14%	77 (62.1)	124 (51.9)	110 (53.7)	0.166
>14%	47 (37.9)	115 (48.1)	95 (46.3)	
Unknown	142	394	144	
Type of histology				
Ductal infiltrating	195 (73.9)	456 (72)	258 (75.2)	<0.001
Ductal in situ	35 (13.3)	61 (9.6)	12 (3.5)	
Lobular	23 (8.7)	71 (11.2)	38 (11.1)	
Other	11 (4.2)	45 (7.1)	35 (10.2)	
Unknown	2	0	6	
Family history				
Yes	19 (12.6)	67 (14.8)	21 (9.5)	0.164
No	132 (87.4)	387 (85.2)	201 (90.5)	
Unknown	115	179	127	
Degree of differentiation				
Category I	48 (34.8)	135 (30)	51 (17.9)	<0.001
Category II	62 (44.9)	190 (42.2)	107 (37.5)	
Category III	28 (20.3)	125 (27.8)	127 (44.6)	
Unknown	128	183	64	
Recurrence				
No	244 (92.1)	566 (89.6)	272 (77.9)	<0.001
Yes	21 (7.9)	66 (10.4)	77 (22.1)	
Death				
No	245 (92.1)	570 (90)	272 (77.9)	<0.001
Yes	21 (7.9)	63 (10)	77 (22.1)	

No significant differences were detected among the three groups in terms of the distribution of comorbidity according to the CCI. After a median follow‐up of 102 months, 22.1% of the interval group, 10.4% of the incident group, and 7.9% of the prevalent group had died (*P* < .001).

The multivariate Cox regression analysis showed that the comorbidity variables according to the CCI, the triple‐negative phenotype, HER2‐enriched subtype, and Stage III were all associated with a higher risk of recurrence and death. However, no such association was observed for an increased histological grade (see Table 2).

**Figure 1:**
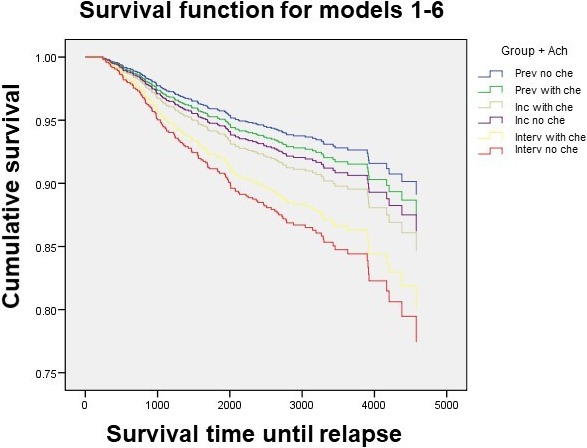
Disease‐free survival of breast cancer patients according to the groups of screening

**Figure 2:**
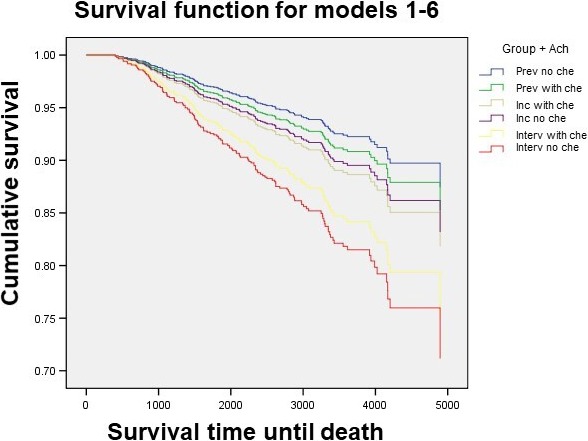
Overall survival according to groups of screening

For the independent principal variable (ACh: yes/no), differences in the proportion of recurrences were observed, but without reaching the statistical significance. However, there were significant differences in overall survival, with a higher risk of death for the interval group without ACh: hazard rate 2.5 (1.0‐6.2) with respect to the other groups. However, for the patients with prevalent or incident cancer who did not receive ACh, there was no greater risk of death. Figures 1 and 2 illustrate the analyses of recurrence‐free survival and overall survival, for each of the patient's groups diagnosed by screening.

**Table 2:** Cox regression analysis adjusted for ACh, Stage, and Charlson comorbidity index (CCI)

	Recurrence		Death	
	HR	aHR^a^	HR	aHR^a^
Group				
Prevalent	Ref.	—	Ref.	—
Incident	1.4 (0.9‐2.4)	—	1.4 (0.9‐2.3)	—
Interval	3.3 (2.0‐5.3)	—	3.5 (2.1‐5.7)	—
Group + Ach				
Prevalent, no chemotherapy	Ref.	Ref.	Ref.	Ref.
Prevalent, with chemotherapy	2.4 (0.9‐6.0)	1,2 (0,4‐3,4)	1.9 (0.7‐4.6)	1,2 (0,4‐3,3)
Incident, with chemotherapy	3.1 (1.4‐7.0)	1,4 (0,6‐3,6)	2.5 (1.1‐5.4)	1,5 (0,6‐3,6)
Incident no chemotherapy	1.9 (0.8‐4.3)	1,4 (0,6‐3,6)	1.7 (0.8‐3.7)	1,4 (0,6‐3,2)
Interval, with chemotherapy	5.0 (2.3‐11.2)	1,9 (0,8‐4,9)	4.5 (2.1‐9.7)	2,1 (0,9‐5,1)
Interval, no chemotherapy	6.4 (2.9‐14.6)	2,2 (0,9‐5,7)	6.1 (2.8‐13.3)	2,5 (1,0 −6,2)
Age				
	1.01 (0.98‐1.04)	—	1.02 (0.99‐1.05)	—
Comorbidity				
Absent	Ref.	—	Ref.	—
Present	1.1 (0.78‐1.56)	—	1.43 (1.03‐1.99)	—
CCI				
	1.0 (0.93‐1.13)	1.1 (0.9‐1.2)	1.1 (1.01‐1.20)	1.1 (1‐1.2)
Phenotype				
Luminal A	Ref.	Ref.	Ref.	Ref.
Luminal B	1.2 (0.8‐1.9)		1.0 (0.6‐1.6)	
Her2	3.2 (1.9‐5.3)		2.8 (1.7‐4.6)	
Triple negative	2.2 (1.3‐3.7)		2.0 (1.2‐3.4)	
Stage				
I	Ref.	Ref.	Ref.	Ref.
IN SITU	0.3 (0.1‐1.0)	0,2 (0,1‐1)	0.2 (0.0‐0.8)	0,2 (0,1‐0,9)
II	1.4 (1.0‐2.2)	1,1 (0,7‐1,8)	1.2 (0.8‐1.8)	0,9 (0,6‐1,5)
III	5.5 (3.7‐8.1)	4,3 (2,7‐6,9)	4.3 (2.9‐6.4)	3,1 (2,0‐4,9)
IV	—		—	
Oestrogen receptors				
Negative	Ref.	—	Ref.	—
Positive	0.6 (0.4‐0.8)	—	0.5 (0.4‐0.8)	—
Progesterone receptors				
Negative	Ref.	—	Ref.	—
Positive	0.6 (0.4‐0.8)	—	0.6 (0.4‐0.8)	—
Her 2				
Negative	Ref.	—	Ref.	—
Positive	1.7 (1.2‐2.5)	—	1.6 (1.1‐2.3)	—
Ki67 expression				
≤14%	Ref.	—	Ref.	—
>14%	1.9 (1.2‐3.0)	—	1.9 (1.2‐3.1)	—
Grade				
I	Ref.	—	Ref.	—
II	2.1 (1.2‐3.7)	—	1.9 (1.1‐3.1)	—
III	3.1 (1.8‐5.4)	—	2.5 (1.5‐4.1)	—
Other	0.7 (0.2‐2.4)	—	0.6 (0.2‐1.9)	—

Note

Sample evaluated: recurrence n = 1.028, Sample evaluated: death n = 1.024.

a Cox regression analysis adjusted for ACh, Stage, and Charlson comorbidity Index.

## 4 DISCUSSION

Screening for breast cancer facilitates detection at an early stage of the disease. Moreover, it is well established that breast cancers detected by screening have a better prognosis, even after adjusting for the stage of the disease.^5^, ^9^, ^13^ In general, these cancers present less aggressive clinical‐pathological and molecular characteristics than those observed in nonscreening or symptomatic patients.^6^, ^14^, ^15^, ^16^ Moreover, differences have also been reported among patients diagnosed during a screening program; patients with interval breast cancer have the poorest prognosis, equal to that of symptomatic patients, due to the larger size of the tumor, the low degree of differentiation, and the presence of lymph node involvement. However, not all studies are in agreement on this question.^17^

However, the use of screening (based on mammography) and the corresponding subgroup (prevalent, incident or interval cancer) is not generally taken into account in stratifying the risk of recurrence and in deciding on the treatment to be provided. In the present study, and unlike previous approaches in this field, classification according to these three subgroups was performed for a large sample of patients, taking part in national public screening programs. Significant differences were observed in the distribution of prognostic factors, with a higher percentage of advanced stages, poorly differentiated, and an absence of hormonal receptors among the patients with interval cancer, followed in frequency by patients with incident and prevalent cancer, respectively. This finding is consistent with and complements the results obtained in a previous pilot study in this respect, in which the tumors diagnosed in the first round presented less aggressive pathological characteristics than those observed among patients with incident cancer.^6^, ^18^

According to previous studies of patients presenting negative results for the expression of hormone receptors, Stages III, grade III histological differentiation and Ki 67 index >14% were positively associated with the likelihood of recurrence and death. A similar association was observed for HER‐2 positive tumors, because adjuvant therapy with trastuzumab, the monoclonal antibody against the HER‐2 receptor, was not yet approved. This treatment option is known to improve the prognosis for this histological subtype.^19^, ^20^

An unavoidable area of bias in our results is the loss of follow‐up for some patients for the analysis of recurrence and death, though this situation only affected a small number of patients.

The patients with incident cancer were at greater risk of recurrence and mortality when the chemotherapy was administered, though the difference disappeared when the stage of the disease and the associated comorbidity were taken into account. Moreover, the presence of interval cancer was associated with poorer rates of survival, especially in the absence of complementary chemotherapy. However, some studies have disputed this conclusion, finding that patients with interval breast cancer diagnosed within 24 months of negative mammographic screening have better survival rates than those diagnosed with no such screening.^21^, ^22^

Comorbidity is a recognized prognostic factor for various tumors and should be taken into account. Moreover, the CCI is a reliable system for evaluating survival, and our study highlights its utility as an independent predictor of mortality in patients with breast cancer diagnosed by screening.^23^, ^24^ Despite these benefits, few previous studies have included this instrument in their evaluation of treatment strategies, and none have examined the influence of ACh in breast cancer tumors identified in national screening programs. One of the main strengths of our study, and hence of the conclusions drawn, is that the survival analysis was adjusted not only for the stage of the disease but also for comorbidity, as determined by the CCI.

Adjuvant chemotherapy has been shown to reduce the probability of recurrence and morbi‐mortality in patients with localized breast cancer. However, when the prognosis is good, this benefit is often minimal and the treatment is not free of side effects, and therefore, clinical or genetic platform is often used to determine the risk of recurrence and to decide whether or not to administer ACh.^25^ To derive a recurrence score for this purpose, these methods combine the classical characteristics of clinical prognosis with the genetic information about the tumor. However, they do not include the information on which the diagnosis was based, which as we see appears to have an independent prognostic value.^26^ This omission could lead to the benefits of systemic treatments being overestimated for the population taking part in the screening program.^8^

In view of these considerations, and having found that the patients with interval breast cancer without the adjuvant treatment have poorer survival rates than the other groups, and that the administration of chemotherapy did not influence the survival of patients with prevalent or incident tumor, we emphasize the importance of considering not only comorbidity but also the origin of the data obtained (ie, from a screening program), when making decisions in usual clinical practice.

## CONFLICT OF INTEREST

None declared.
